# A curated dataset of multi-channel TV program schedules for optimization and benchmarking

**DOI:** 10.1016/j.dib.2026.112568

**Published:** 2026-02-09

**Authors:** Kadri Sylejmani, Shefket Bylygbashi, Uran Lajçi, Zenun Kastrati

**Affiliations:** a Faculty of Electrical and Computer Engineering, University of Prishtina, Bregu i Diellit p.n, 10000 Prishtina, Kosova; b Department of Informatics, Linnaeus University, Universitetsplatsen 1, 351 95, Växjö, Sweden

**Keywords:** Real-world data, Broadcast planning, Constraint satisfaction, Time windows, Penalty functions, Genre composition, Metaheuristic search, Dimensionality reduction

## Abstract

This article presents a collection of 515 Smart TV scheduling instances from three public sources: IPTV EPG broadcast listings, EPG.PW program guides, and upcoming YouTube livestream schedules obtained through the YouTube Data API. The data were collected and processed using automated Python scripts that download program information, convert it into a standard format, and remove entries with missing or invalid time information. All times are recorded as minutes from the start of each scheduling period to ensure consistency across different sources.

Each instance is stored as a JSON file with a consistent structure. Each file contains the scheduling period (start and end times), channel information, program time slots that don't overlap, program categories, and quality scores from 0 to 100 for each program. Instances also include two types of constraints: strict rules that specify which channels must be available during certain time windows, and flexible preferences that assign bonus points to programs airing at preferred times. All files are organized by data source and automatically checked for correctness.

The dataset can be used for benchmarking optimization and constraint-based scheduling methods, comparing objective formulations with switching and termination penalties, testing algorithms under time-dependent feasibility constraints, and extracting instance features for dataset characterization and dimensionality-reduction workflows.

Specifications Table

**Table:** 

Subject	Computer Sciences
Specific subject area	Benchmark datasets for smart TV scheduling, broadcast optimization, and constraint-based media planning.
Type of data	Structured scheduling instances (JSON). Processed, filtered, normalized, and annotated data.
Data collection	The dataset was generated using automated Python-based data collection and transformation pipelines. Television broadcast schedules were collected from publicly available IPTV Electronic Program Guide (EPG) feeds and the EPG.PW XMLTV service, which provide structured program listings for multiple countries. In addition, metadata for announced upcoming livestreams were retrieved using the YouTube Data API v3. The collected data were filtered by target date, minimum program duration, and channel validity. All sources were normalized into a unified instance-based JSON format with consistent temporal indexing, non-overlapping programs, inferred genre labels, priority constraints, and time-preference annotations suitable for scheduling and optimization experiments.
Data source location	IPTV Electronic Program Guide data were collected from publicly available international XMLTV feeds hosted at *iptv-epg.org*. EPG.PW data were obtained from publicly accessible XMLTV feeds provided by *epg.pw*. YouTube livestream metadata were retrieved via the YouTube Data API v3 using region- and language-specific queries. All processed data are stored at the authors’ institutional affiliation and publicly hosted in an online repository.
Data accessibility	All data associated with this article are publicly available. Repository name: Smart TV Scheduling Dataset Data identification number: https://zenodo.org/records/18284422 Direct URL to data: https://github.com/shefketbylykbashi/smart-tv-scheduling-dataset Instructions for accessing the data: All instances are provided in JSON format and organized by source under the Instances/ directory. No authentication is required to access or download the dataset.
Related research article	None

## 1 Value of the Data

• This dataset provides realistic, time-indexed multi-channel television schedules where each channel contains temporally sequenced programs with start/end times, genre classifications, and utility scores. The data structure directly supports research in automated scheduling for public venues (e.g., waiting rooms, cafeterias, gyms) where a single display must be optimized across available broadcast options throughout the operating day.
• All instance-level parameters required for reproducible optimization experiments are embedded within the dataset, including venue operating hours, minimum program display durations, genre repetition limits, and switching/termination penalty coefficients. Researchers can utilize these standardized instances without introducing external assumptions, enabling direct comparison of scheduling algorithms and objective functions across studies.
• Time-dependent operational constraints are encoded through priority time blocks that restrict channel eligibility during specific intervals. These constraints reflect real-world requirements such as contractual obligations, content policies, or event-driven restrictions, allowing researchers to evaluate scheduling approaches under dynamic feasibility conditions that vary throughout the day.
• The dataset includes explicit time-of-day preference specifications, defining genre bonuses within designated intervals (morning, afternoon, evening). This structured representation enables research in context-aware scheduling where content desirability varies temporally, supporting studies in preference-based optimization, viewer satisfaction modelling, and time-sensitive content recommendation.
• The collection encompasses diverse instance configurations varying in channel count, operating windows, genre distributions, and constraint parameters. This heterogeneity enables benchmarking of solution methods across different public-venue scenarios. The consistent JSON format facilitates automated feature extraction for dataset characterization, statistical analysis, and dimensionality reduction techniques.
• The dataset's rich temporal and categorical features (start/end times, genres, utility scores across channels) enable machine learning applications including predictive modeling and recommender systems. Models can be trained to forecast viewer preferences, predict program engagement, or recommend personalized content based on scheduling patterns. This supports data-driven advances in TV analytics, audience prediction, and intelligent content delivery beyond traditional optimization.
• The utility scores are synthetic values designed for optimization benchmarking, not empirical viewership ratings or Nielsen-style metrics [1]. These heuristic scores combine domain knowledge with controlled randomization to create realistic scheduling scenarios while ensuring reproducibility and avoiding privacy concerns. Researchers can substitute real-world viewership data when available while preserving the dataset's temporal and constraint structure.

## 2 Background

This dataset was compiled to support research in combinatorial optimization and automated decision-making for public venue management [2]. The motivation stems from a practical challenge faced by cafes, lounges, gyms, and waiting areas that must select television content from multiple broadcast channels to maximize viewer satisfaction throughout their operating hours. Research on situated/public displays highlights that coordinating what is shown, when it is shown, and under what constraints is a non-trivial scheduling problem in real deployments [3].

Traditional approaches in venues often rely on manual selection or static rules, which do not capture time-dependent context or operational requirements. In contrast, the broader literature on context-aware systems and recommenders shows that incorporating context such as time-of-day can improve relevance of content choices, motivating optimization formulations that explicitly represent temporal preferences [4].

The dataset formalizes the venue content-selection task as a constrained optimization problem where venues balance competing objectives: maximizing content utility (e.g., popularity scores) and time-of-day preference bonuses while minimizing penalties associated with frequent switching and interruptions. Related work in television scheduling has long modelled scheduling decisions as optimization problems that aim to improve audience outcomes under practical constraints, including early analytical and mathematical programming formulations for TV program scheduling [5]. In addition, research on digital signage and public display scheduling has proposed utility-based and context-sensitive scheduling frameworks for determining what content should be shown on shared screens in public environments, reinforcing the need for standardized, machine-readable instances to evaluate scheduling methods under realistic constraints [6].

By providing standardized instances with embedded parameters and constraints, this dataset enables reproducible benchmarking of optimization algorithms, heuristic methods, and machine learning approaches in venue-based content scheduling - a domain that bridges operations research, recommender systems, and context-aware computing. The consistent JSON format facilitates automated processing and integration into existing optimization and experimentation pipelines.

## 3 Data Description

**Fig. 1:**
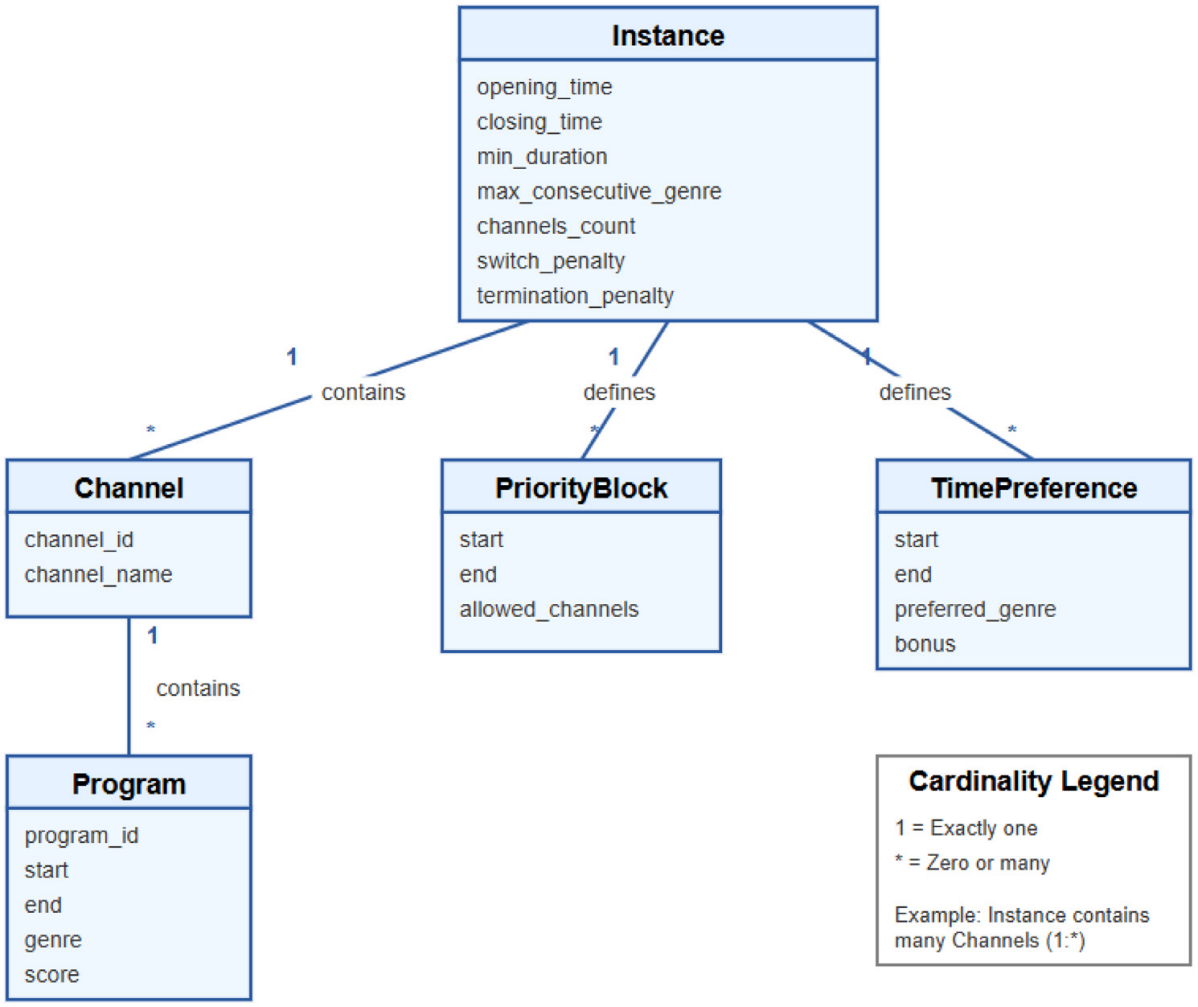
Conceptual schema of a Smart TV scheduling instance.

The dataset consists of standardized instances of the Smart TV scheduling problem, constructed according to a single, unified JSON schema. All instances share a common structural representation, enabling direct comparison across different problem configurations and ensuring methodological consistency for benchmarking scheduling and optimization algorithms. Fig. 1 illustrates the conceptual schema of a Smart TV scheduling instance. Each instance defines a global scheduling horizon and includes a set of channels, each with an associated sequence of programs.

Table 1 summarizes the complete set of parameters used to describe an instance, including global scheduling parameters, channel and program attributes, and constraint definitions. The complete formal JSON Schema enforcing structural validity is publicly available in the repository (Metadata/instance_schema.json) and supports automatic validation of all instances.

**Table 1:** Summary of attributes used in a Smart TV scheduling instance.

Attribute	Type	Description
*opening_time*	Integer	Start of the scheduling horizon, expressed in minutes from the beginning of the instance timeline.
*closing_time*	Integer	End of the scheduling horizon (exclusive), in minutes.
*min_duration*	Integer	Minimum allowed duration of a program (in minutes).
*max_consecutive_genre*	Integer	Maximum number of consecutive programs of the same genre allowed before penalties apply.
*channels_count*	Integer	Total number of channels included in the instance.
*switch_penalty*	Integer	Penalty applied when switching between channels in consecutive scheduled segments.
*termination_penalty*	Integer	Penalty applied when the schedule terminates before fully covering the scheduling horizon.
*channels.channel_id*	Integer	Unique numeric identifier assigned to each channel.
*channels.channel_name*	String	Human-readable name of the channel.
*programs.program_id*	String	Unique identifier of a program within its channel.
*programs.start*	Integer	Program start time in minutes (inclusive) relative to the instance timeline.
*programs.end*	Integer	Program end time in minutes (exclusive).
*programs.genre*	String	Genre or category assigned to the program.
*programs.score*	Integer (0–100)	Program utility or quality score used by optimization algorithms.
*priority_blocks.start*	Integer	Start time of a priority block (inclusive), in minutes.
*priority_blocks.end*	Integer	End time of a priority block (exclusive), in minutes.
*priority_blocks.allowed_channels*	Integer array	Set of channel identifiers allowed to be scheduled during the priority block.
*time_preferences.start*	Integer	Start time of a time preference window (inclusive), in minutes.
*time_preferences.end*	Integer	End time of a time preference window (exclusive), in minutes.
*time_preferences.preferred_genre*	String	Genre preferred during the specified time window.
*time_preferences.bonus*	Integer	Bonus added to the objective function when the preferred genre is scheduled in the corresponding time window.

All instances are organized into three main groups, according to the origin of the underlying data source: IPTV EPG, EPG.PW, and YouTube Upcoming Livestreams.

### 3.1 Dataset organization by data source

#### 3.1.1 Instances derived from IPTV EPG and EPG.PW

For instances generated from IPTV EPG and EPG.PW, the core instance structure includes a fixed set of global scheduling parameters: opening_time, closing_time, min_duration, max_consecutive_genre, switch_penalty, and termination_penalty. These parameters are author-defined and constant across all IPTV and EPG.PW instances, ensuring comparability. The scheduling horizon spans exactly one day, with min_duration = 30 min, max_consecutive_genre = 2, switch_penalty = 10, and termination_penalty = 20.

For these instances, priority blocks are constructed as hard temporal constraints that restrict which channels may be scheduled during specific time intervals. The construction process is fully deterministic and proceeds as follows:

• The scheduling horizon is partitioned into fixed 15-minute intervals.
• For each interval, a score mass is computed by aggregating the overlap duration of all programs with that interval, weighted by their respective program scores.
• Intervals with high cumulative score mass (representing peak content concentration) are identified as critical intervals.
• Adjacent critical intervals are merged into priority blocks of minimum duration 30 min.
• For each priority block, dominant channels are identified as those contributing most to the total score within the block.
• The set of allowed channels is defined as the smallest subset whose cumulative contribution accounts for at least 60 % of the block’s total score.

Time preferences are generated as soft constraints according to the following procedure:

• The scheduling horizon is divided into equal temporal segments of at least 30 min.
• For each segment, all overlapping programs are analysed.
• For each genre, a cumulative weight is computed based on program duration and score.
• The genre with the highest cumulative weight is selected as the preferred genre for that segment.
• A bonus value is assigned proportionally to the genre’s dominance and the temporal importance of the segment (e.g., higher bonuses during prime-time).

Channel and program data are mapped directly from the original XMLTV feeds provided by IPTV EPG and EPG.PW. Program start times, end times, and durations are preserved without modification.

Program genres are inferred using an advanced multilingual heuristic, combining:

• XMLTV category tags,
• lexical analysis of program titles and descriptions,
• multilingual genre dictionaries.

Program scores are computed deterministically by combining:

• a base genre weight,
• program duration,
• temporal position within the day,
• presence of signaling keywords,
• and a small, channel-specific, stable pseudo-random component.

#### 3.1.2 Instances derived from YouTubeupcoming livestreams

Instances derived from YouTube Upcoming Livestreams are synthetically generated but grounded in real future livestream metadata obtained via the YouTube Data API v3. Each upcoming livestream is modelled as a program, while collections of livestreams are aggregated into virtual television channels.

For YouTube-based instances:

• The scheduling horizon varies between 1 and 20 days, enabling both short-term and long-term planning scenarios.
• The minimum program duration is lower than in IPTV/PW instances, reflecting the more dynamic and irregular nature of livestream content.
• Parameters such as max_consecutive_genre, switch_penalty, termination_penalty, priority blocks, and time preferences are generated parametrically, producing instances with controlled and varying levels of difficulty.

Priority blocks in YouTube instances are constructed to emulate high-attention livestream windows, such as major esports tournaments, live news coverage, or music events. Dominant channels are selected based on cumulative livestream relevance and expected viewer impact.

Time preferences are generated using genre dominance and temporal relevance across the extended planning horizon, with higher bonuses assigned to periods with dense concentrations of high-impact livestreams.

Program genres and scores are inferred from livestream titles, descriptions, topics, and contextual metadata using the same multilingual genre inference framework applied to IPTV and EPG.PW. The number of programs per channel ranges from 5 to 30, simulating channels with diverse content densities and heterogeneous scheduling complexity.

This unified representation across three distinct data sources enables systematic evaluation of scheduling algorithms under realistic, large-scale, and structurally diverse conditions, while maintaining full reproducibility and formal consistency.

## 4 Technical Validation

To characterize dataset heterogeneity, we provide several complementary visualizations including diversity analyses and instance space characterizations using established statistical metrics and dimensionality reduction techniques.

### 4.1 Visualization of dataset diversity

This section summarizes differences in instance scale, constraint structure, temporal occupancy, program duration, and hard-constraint strictness across the three data sources (IPTV EPG, EPG.PW, and YouTube Upcoming Livestreams).

#### 4.1.1 Instance-scale diversity

The overall scale of each scheduling instance is characterized by the number of channels and the total number of programs contained within the scheduling horizon. This representation captures heterogeneity in instance size and content density across data sources.

**Fig. 2:**
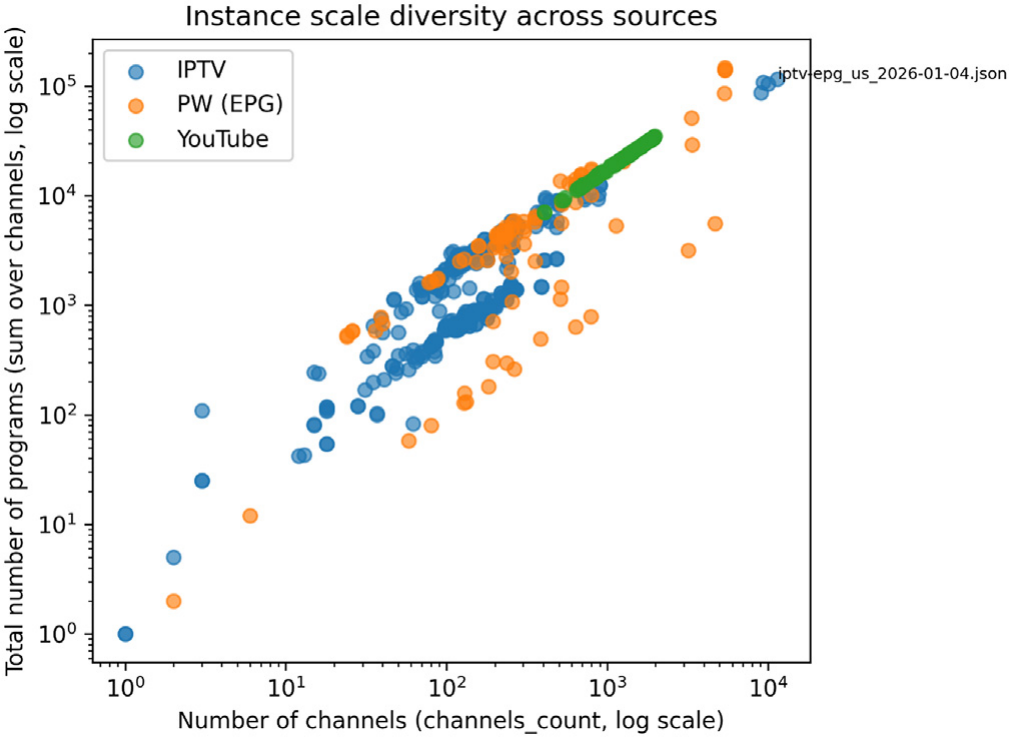
Distribution of instance sizes across data sources, measured by number of channels and total number of programs.

In Fig. 2, each point corresponds to a single instance, plotted by its channel count and total number of programs. Instances derived from IPTV EPG and EPG.PW are characterized by high channel counts and dense program populations, reflecting structured broadcast schedules. YouTube-derived instances occupy a distinct region, exhibiting comparable channel counts but greater variability in program density due to the irregular nature of livestream content.

#### 4.1.2 Temporal occupancy diversity

Temporal diversity is assessed by analysing how channel activity is distributed over time within each instance. Program schedules are aggregated into fixed 30-minute time bins, and the number of active channels per bin is computed.

**Fig. 3:**
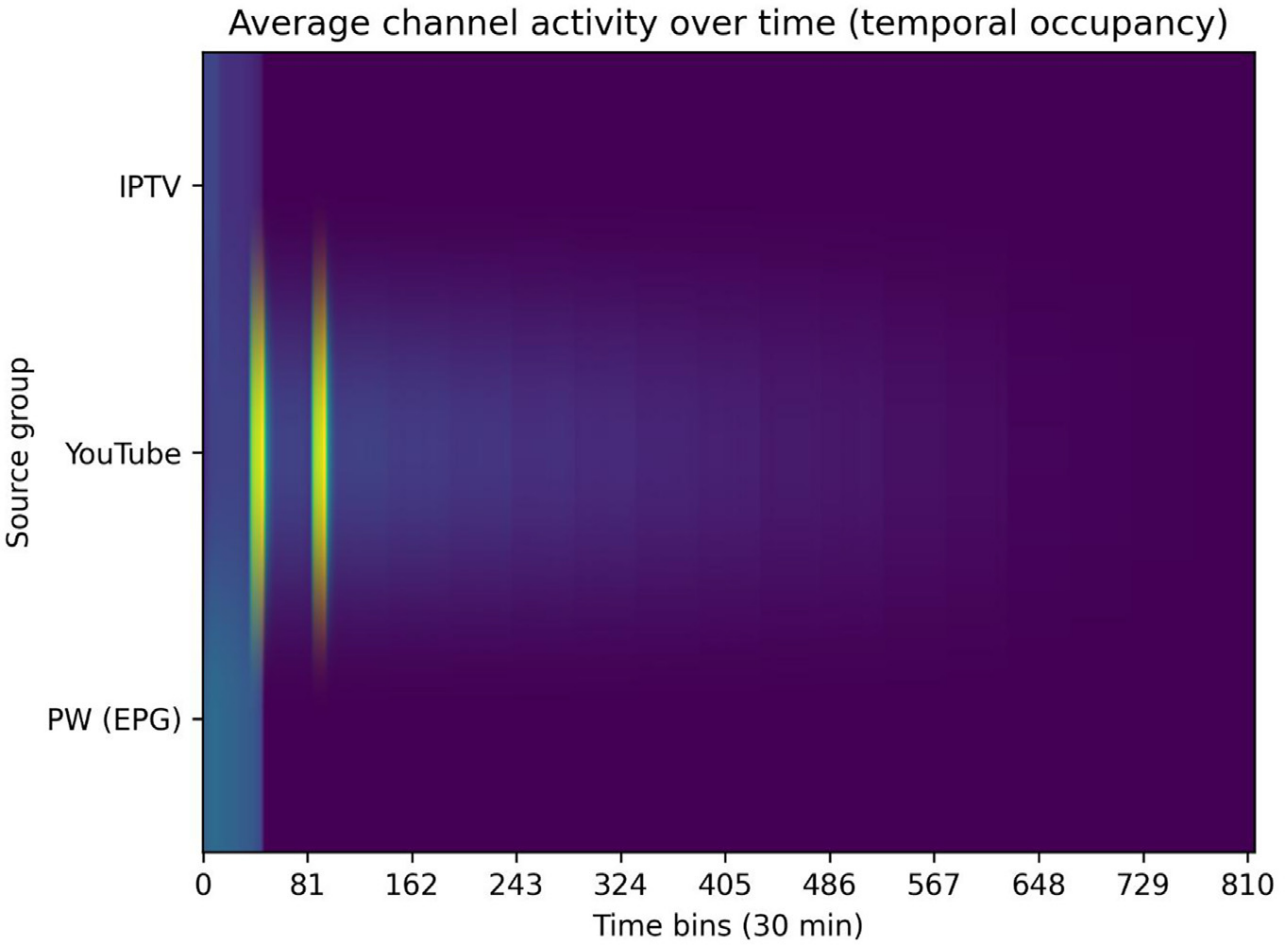
Average channel occupancy profiles by data source, computed using 30-minute time bins.

In Fig. 3, occupancy profiles are averaged across instances belonging to the same data source. IPTV EPG and EPG.PW instances exhibit relatively stable and uniform occupancy patterns across the scheduling horizon, reflecting regular broadcast structures. In contrast, YouTube-derived instances show highly concentrated activity in the early time bins, followed by a rapid decline, indicating irregular and front-loaded scheduling behaviour. The figure highlights clear differences in temporal density and schedule regularity across heterogeneous data sources.

#### 4.1.3 Program duration diversity

The dataset also exhibits variability in temporal granularity, captured through program duration distributions. Program duration is defined as the difference between program end and start times.

**Fig. 4:**
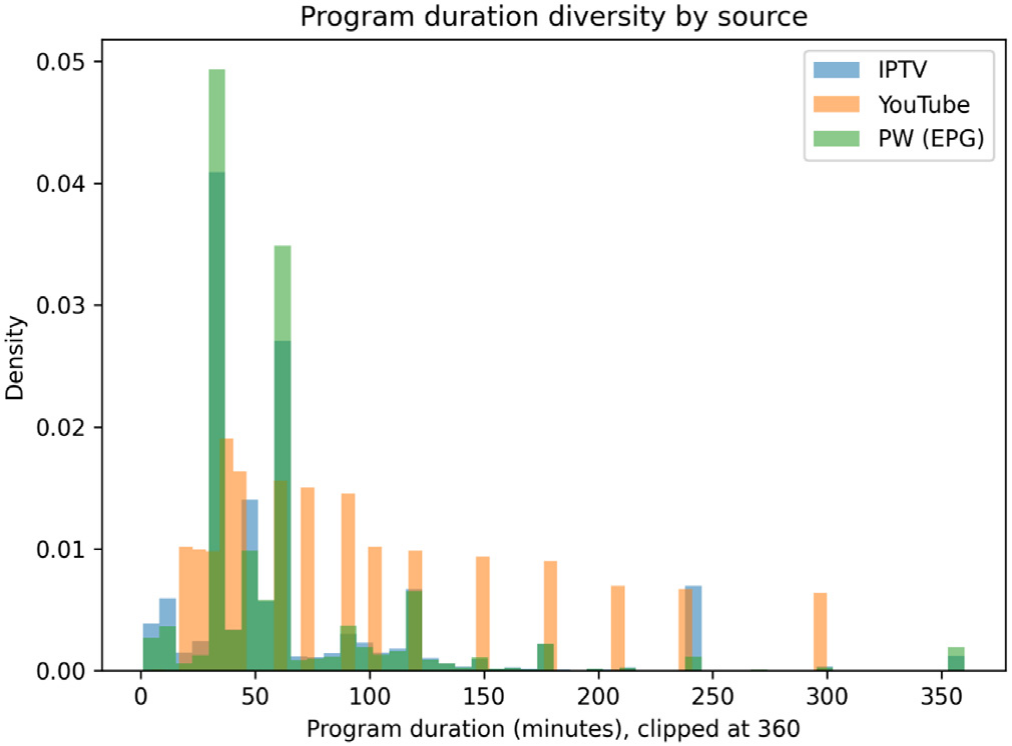
Normalized distributions of program durations by data source.

Fig. 4 shows clear source-specific duration patterns. EPG.PW (green) has very sharp peaks around 30 min and 60 min, indicating highly standardized slot lengths, with only a thin tail and rare very long programs (approaching the 360-minute clip). IPTV (blue) is also concentrated around 30–60 min, but exhibits a more visible tail, including occasional long durations around 240 min. In contrast, YouTube (orange) spreads much more broadly: while it still has mass around 30–60 min, it shows substantial density across ≈ 70–200 min and clear outliers up to ≈ 300 min, consistent with heterogeneous livestream/event durations. Overall, the diagram highlights much finer and more regular segmentation in broadcast EPG sources versus more variable temporal granularity in YouTube-derived schedules.

#### 4.1.4 Constraint strictness diversity

Hard-constraint strictness is quantified using the priority block coverage ratio, defined as the fraction of the scheduling horizon covered by priority blocks. To avoid double counting, overlapping priority blocks are merged before computing the total covered duration.

**Fig. 5:**
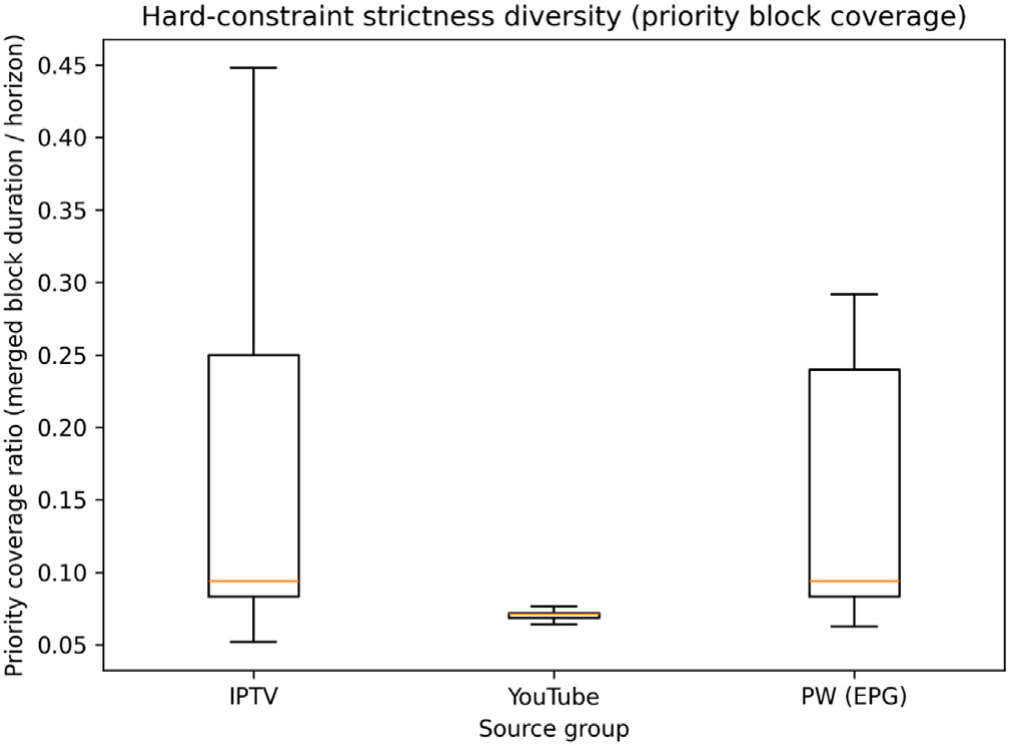
Distribution of priority block coverage ratios across instances by data source.

Fig. 5 reveals distinct source-dependent patterns in hard-constraint strictness. YouTube instances exhibit consistently low priority block coverage (median ≈ 0.07) with minimal dispersion, indicating that channel restrictions occupy a small and relatively stable fraction of the scheduling horizon. In contrast, IPTV and EPG.PW instances display substantially greater variability: while both sources share similar medians (≈ 0.09), their distributions are considerably wider, with the third quartile reaching approximately 0.24–0.25 and outliers extending to 0.45 for IPTV and 0.29 for EPG.PW, respectively. This heterogeneity suggests that YouTube-derived instances present more uniform constraint landscapes, whereas IPTV and EPG.PW instances span a broader spectrum of scheduling difficulty, with some configurations imposing substantially tighter temporal restrictions on channel availability.

#### 4.1.5 Constraint interaction complexity

Hard constraints vary not only in their temporal extent but also in their degree of channel restrictiveness. To jointly characterize these orthogonal dimensions, we introduce a two-dimensional constraint interaction framework that maps instances according to their temporal coverage and channel selectivity.

**Fig. 6:**
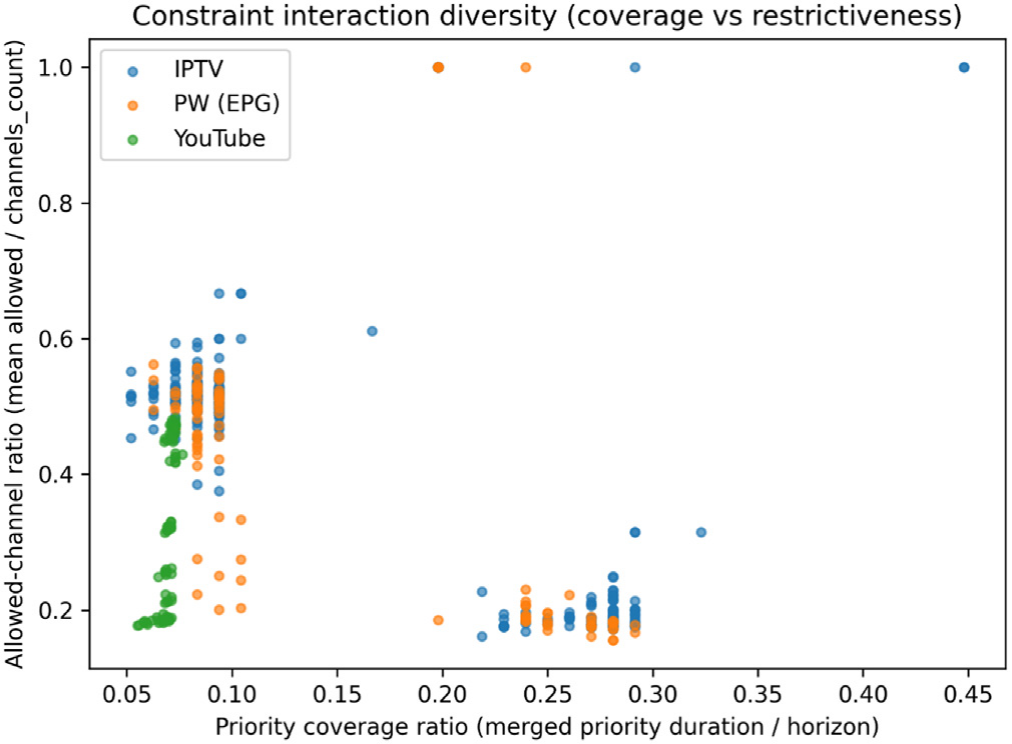
Joint distribution of merged priority-block coverage ratio and allowed-channel restrictiveness ratio, summarizing diversity in hard-constraint regimes across instance sources.

Fig. 6 reveals distinct clustering patterns that characterize the constraint landscape across data sources. The majority of instances concentrate in a low-coverage regime (coverage ratio ≈ 0.05–0.10) with moderate channel restrictiveness (allowed-channel ratio ≈ 0.45–0.55), indicating temporally sparse constraints that nonetheless restrict approximately half of available channels. A secondary, well-separated cluster emerges in the high-coverage regime (≈ 0.22–0.30) with substantially greater restrictiveness (allowed-channel ratio ≈ 0.17–0.23), representing instances where priority blocks span extended temporal intervals while permitting only a limited channel subset. Further, source-specific patterns are evident in the spatial distribution of instances. YouTube-derived instances predominantly occupy the low-coverage region, with several exhibiting notably low allowed-channel ratios (≈ 0.17–0.30) despite limited temporal extent - a configuration that imposes strict channel restrictions over brief intervals. Conversely, IPTV and EPG.PW instances span both regimes, with significant representation in the high-coverage, highly restrictive quadrant. Several isolated outliers demarcate the boundary cases: instances with minimal restrictiveness (allowed-channel ratio ≈ 1.0) where priority blocks impose negligible channel constraints, and instances with extreme temporal coverage (up to ≈ 0.45) representing near-continuous constraint enforcement throughout the scheduling horizon.

#### 4.1.6 Scale inequality and heavy-tailed structure

Instance scale diversity is further characterized using inequality and tail-distribution analyses. This representation captures the presence of heavy-tailed behaviour and unequal instance sizes, which are relevant for stress-testing scheduling algorithms.

**Fig. 7:**
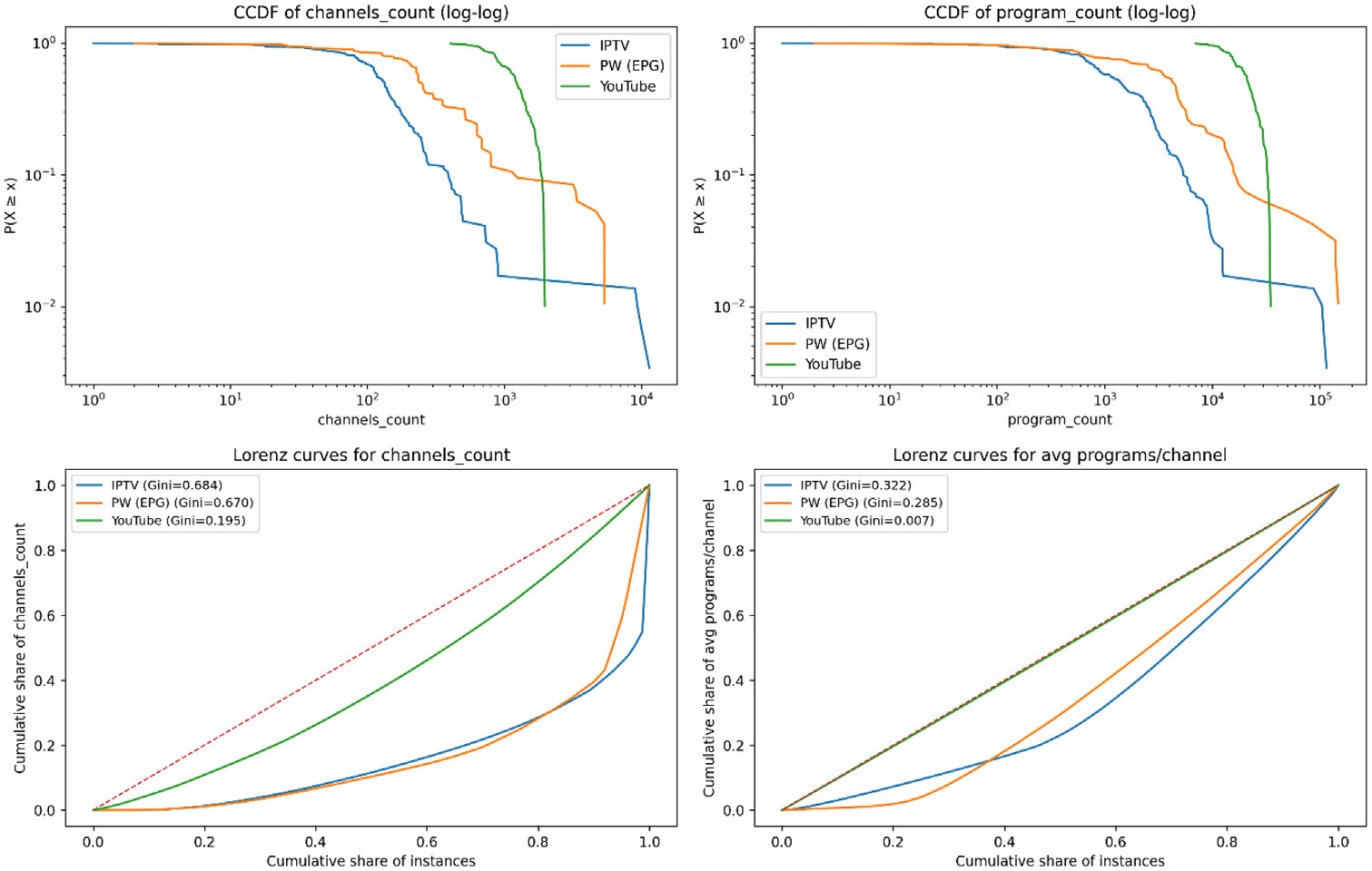
Scale inequality and heavy-tail structure across sources, shown using CCDFs, Lorenz curves, and Gini coefficients.

Fig. 7 combines distributional and inequality-based views of instance scale. In the log-log CCDFs, YouTube-derived instances (green) are shifted farthest to the right, indicating substantially larger typical sizes, with channel counts concentrated around the 10³ range and program counts extending toward 10⁴−10⁵. IPTV and EPG.PW occupy lower ranges overall, but both exhibit heavy tails: EPG.PW (orange) retains a larger probability mass at high values and reaches the largest extremes (≈ 10⁴ channels and ≈ 10⁵ programs), while IPTV (blue) decays earlier and shows fewer very large instances.

The Lorenz curves quantify how unevenly instance sizes are distributed. Channel counts are highly unequal for IPTV and EPG.PW (Gini 0.684 and 0.670), meaning a small fraction of instances accounts for a large share of channels, whereas YouTube is much more balanced (Gini 0.195). For average programs per channel, inequality is moderate for IPTV and EPG.PW (Gini 0.322 and 0.285) but is near uniform for YouTube (Gini 0.007), with its Lorenz curve almost overlapping the equality line. Overall, the figure shows that broadcast EPG sources combine strong scale heterogeneity with pronounced right tails, while YouTube instances are consistently large yet comparatively homogeneous, especially in programs-per-channel.

#### 4.1.7 Temporal activity structure and entropy

Temporal structure diversity is assessed by analysing how program activity is distributed over the scheduling horizon. This captures whether schedules are uniformly populated or concentrated within specific time windows. For each instance, program intervals are aggregated into fixed-duration time bins, and channel-level occupancy is computed per bin. Source-level activity profiles are obtained by averaging these occupancy patterns across instances. In addition, normalized Shannon entropy is computed per instance to quantify temporal concentration (low entropy) versus uniform coverage (high entropy).

**Fig. 8:**
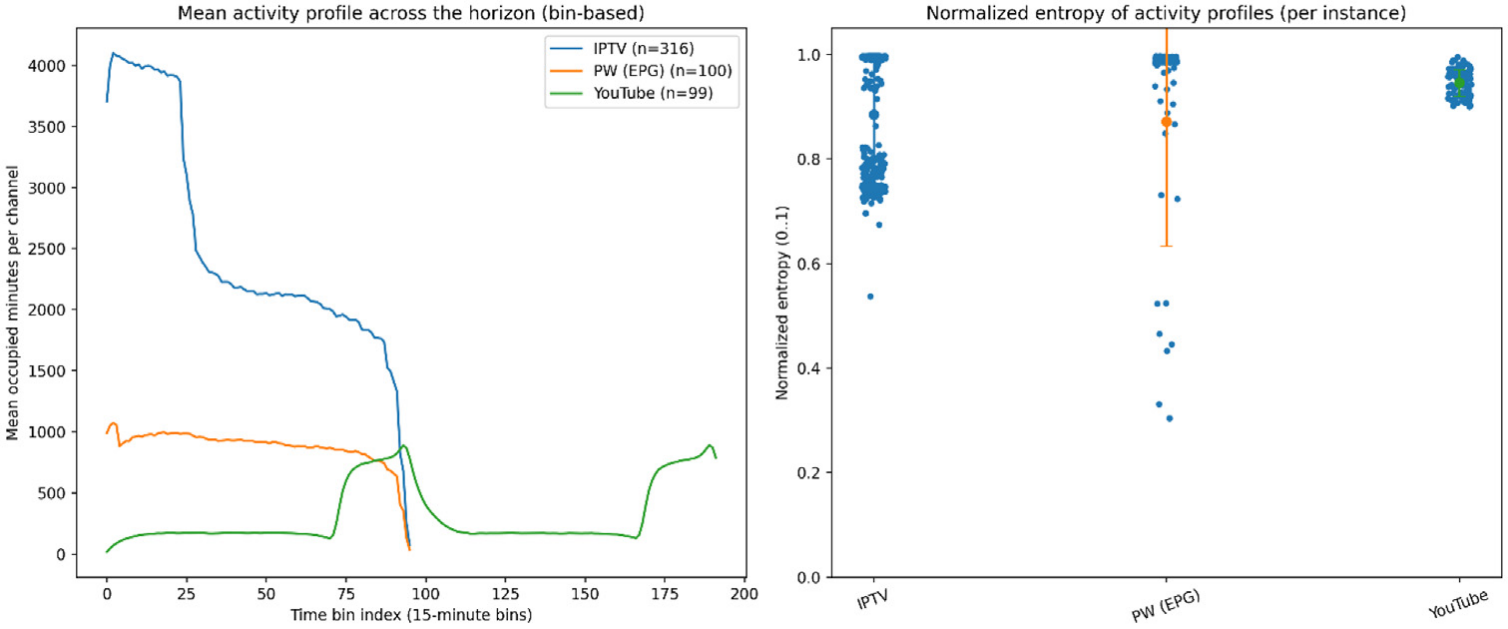
Temporal activity structure and normalized entropy across sources.

Fig. 8 compares sources using both the mean activity profile (15-minute bins) and per-instance normalized entropy. IPTV shows the highest, clearly front-loaded occupancy, declining steadily and dropping sharply near the end. EPG.PW follows a similar but lower and smoother pattern, also decreasing toward the end. YouTube differs markedly, with generally low activity punctuated by step-like bursts and a late increase. Entropy values confirm these differences: YouTube instances cluster tightly at high entropy (≈ 0.92–0.97), indicating consistently uniform temporal spread. IPTV is mostly high-entropy but more dispersed, while EPG.PW has the widest spread, from moderate outliers (≈ 0.3–0.5) up to near 1.0, reflecting mixed temporal regularity.

#### 4.1.8 Content–constraint coupling

Beyond constraint structure alone, the dataset exhibits diversity in how hard constraints interact with content utility. This interaction is characterized by jointly considering constraint restrictiveness, temporal coverage, and score concentration. For each instance, mean priority-block tightness is measured as the fraction of allowed channels within constrained windows, while coverage represents the fraction of the horizon subject to priority blocks. Content interaction is quantified by computing the fraction of total program score mass that lies within constrained intervals. Marker size encodes this score concentration, linking constraint structure to content utility.

**Fig. 9:**
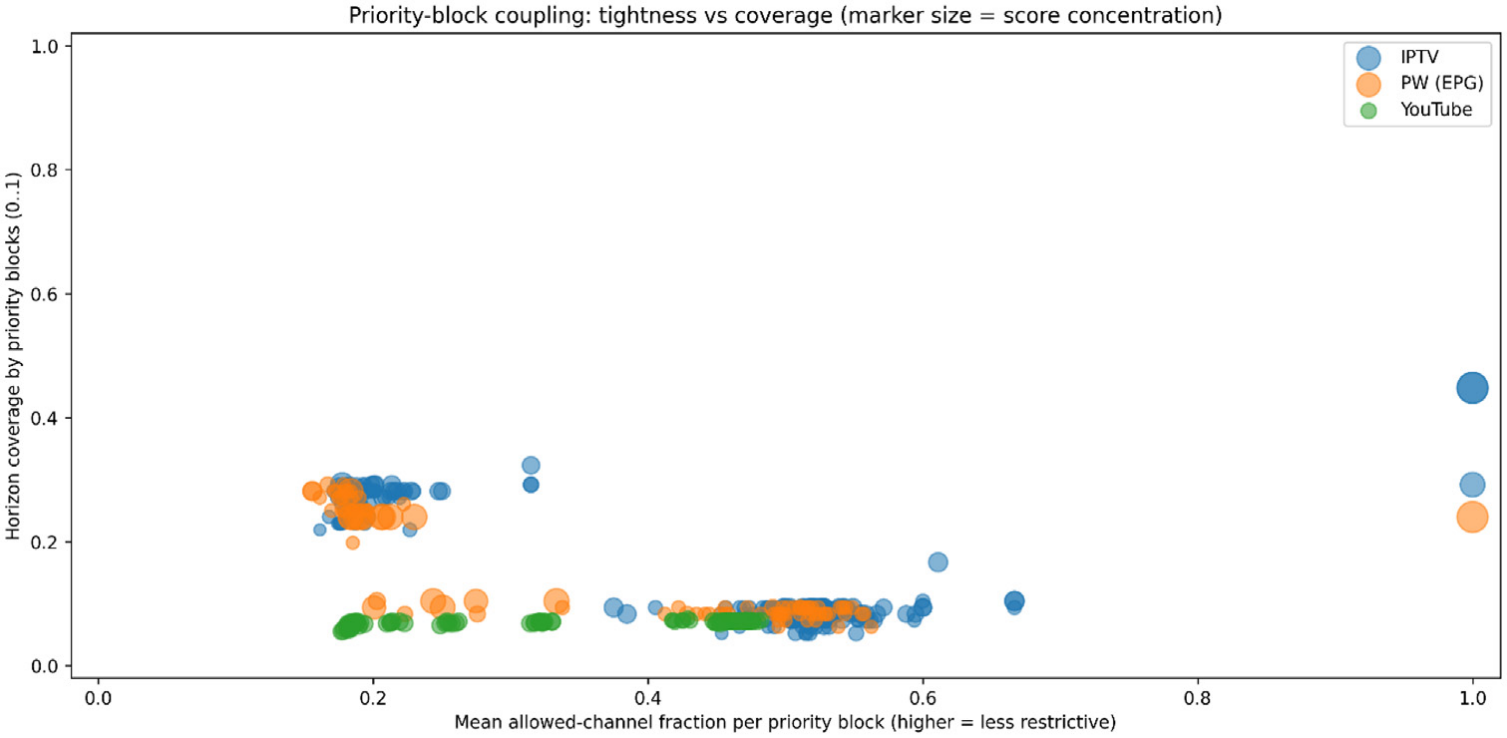
Coupling between hard constraints and content utility across instances.

Fig. 9 maps instances by priority-block restrictiveness (mean allowed-channel fraction) and temporal coverage, with marker size indicating score concentration. Most IPTV and EPG.PW instances cluster at moderate coverage (≈ 0.20–0.30) with low allowed-channel fractions (≈ 0.15–0.25), reflecting tightly coupled, restrictive priority blocks. A second, denser band appears at lower coverage (≈ 0.05–0.10) with higher allowance (≈ 0.40–0.60), indicating shorter but more permissive constraints. YouTube instances are concentrated at very low coverage and low-to-moderate allowance, showing generally light and weakly coupled constraints. A few outliers with near-unrestricted blocks (allowed ≈ 1.0) and high coverage highlight extreme cases.

#### 4.1.9 Conflict graph density and sparsity spectrum

Scheduling difficulty is influenced not only by constraint structure and scale, but also by the degree of temporal overlap among programs. To characterize this dimension of diversity, conflict density metrics are computed for each instance based on concurrent program activity. For each instance, an event-based sweep algorithm is applied over the scheduling horizon. Program start and end times define event points that partition the horizon into disjoint intervals. Within each interval, the number of concurrently active programs is tracked, and the number of pairwise conflicts is computed as the binomial coefficient of active programs. These values are integrated over time to obtain a time-weighted measure of conflict intensity. The resulting conflict mass is normalized by the horizon length and the maximum possible conflicts, yielding a normalized conflict density in the range [0,1]. In addition, summary concurrency statistics, including mean and peak numbers of active programs, are computed.

**Fig. 10:**
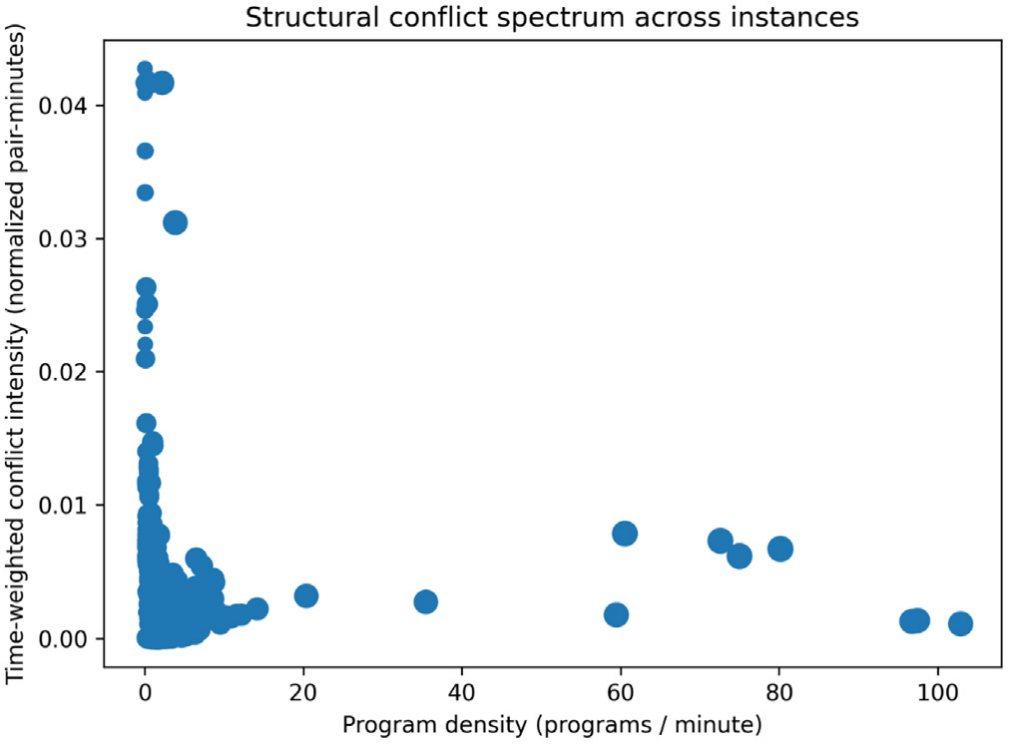
Conflict graph density and sparsity spectrum across instance sources, based on time-weighted concurrent program activity.

Fig. 10 maps instances along a program-density axis (programs/minute) versus time-weighted conflict intensity (normalized pair-minutes), revealing a strongly skewed spectrum: most instances cluster at low program density (<≈ 10 programs/min) with generally low conflict intensity, while a small number of high-density outliers (≈ 60–100 programs/min) remain at moderate conflict levels. A few low-density instances exhibit very high conflict intensity (up to ∼0.04), indicating cases where overlap is structurally concentrated despite small overall scale.

#### 4.1.10 Multi-scale temporal burstiness and fractality

Beyond aggregate occupancy patterns, the dataset exhibits diversity in the fine-scale temporal organization of program start events. To quantify this property, burstiness and fractal scaling analyses are performed on program start-time sequences. For each instance, inter-arrival times between consecutive program starts are computed and summarized using the burstiness index B=(σ−μ)/(σ+μ), where μ and σ denote the mean and standard deviation of inter-arrival times, respectively. Values close to −1 indicate regular timing, while values near 1 indicate strong burstiness. In parallel, detrended fluctuation analysis (DFA) [7] is applied to the binned start-count signal to estimate a scaling exponent that captures long-range temporal correlations and fractal structure.

**Fig. 11:**
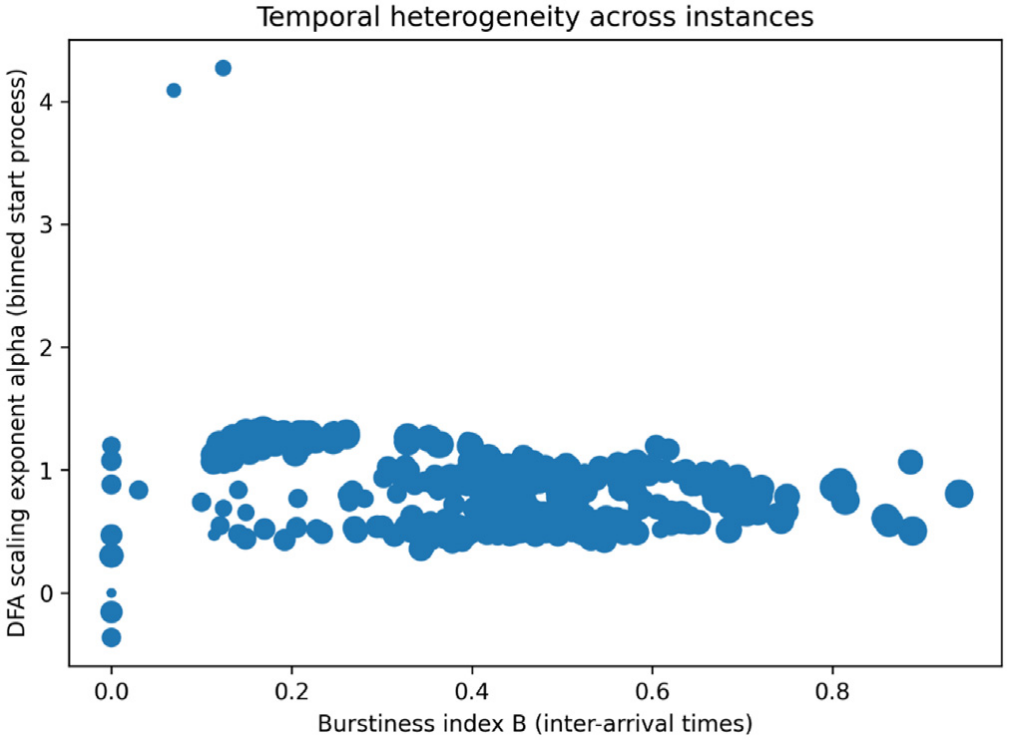
Temporal burstiness and fractal scaling properties of program start times across sources.

Fig. 11 plots each instance by its burstiness index B and DFA scaling exponent α, showing that most instances fall in a broad band with moderate-to-high burstiness (B≈0.3–0.7) and α near 0.5–1.2, indicating a mix of bursty start patterns and weak-to-moderate long-range correlations. The densest region concentrates around B≈0.4–0.6 with α≈0.6–1.0. A small number of outliers appear at very low burstiness (B≈0) and at extreme DFA exponents (including α>4 and a few near or below 0), highlighting rare instances with highly atypical temporal structure.

#### 4.1.11 Constraint–objective tension surface

The interaction between hard constraints, content utility, and penalty structure determines the effective optimization landscape faced by scheduling algorithms. To summarize this interaction, a multidimensional constraint–objective representation is constructed. For each instance, four normalized metrics are computed: (i) hard-constraint coverage ratio, defined as the merged duration of priority blocks divided by the horizon length; (ii) constraint tightness, measured as the weighted mean fraction of allowed channels within priority blocks; (iii) objective potential, defined as the time-weighted mean program score density over the horizon; and (iv) penalty pressure, computed as a normalized combination of switching and termination penalties. Instances are embedded in a two-dimensional plane using hard-constraint coverage and objective potential as axes, while marker size encodes instance scale and marker shading encodes penalty pressure.

**Fig. 12:**
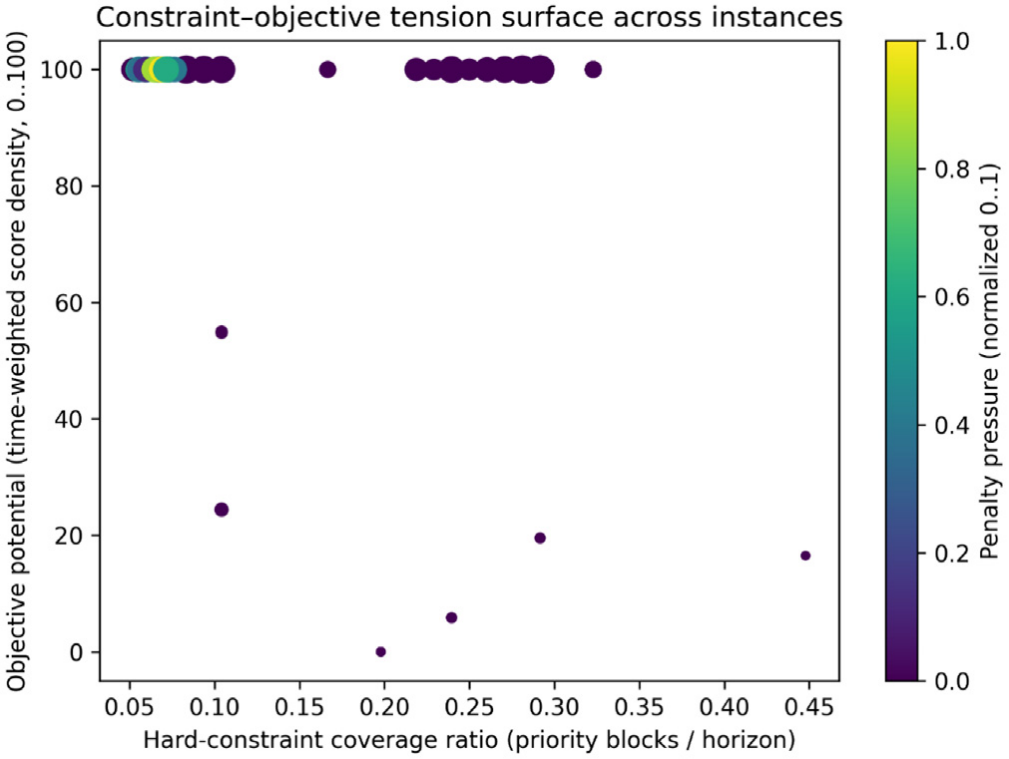
Constraint–objective tension surface summarizing interactions between hard constraints, content utility, and penalty structure.

Fig. 12 shows that most instances cluster along the upper boundary of objective potential (near 100 on the normalized scale) across a wide range of hard-constraint coverage, indicating many cases where high score density is available despite differing levels of enforced priority blocks. A dense group appears at low coverage (≈ 0.05–0.10) with predominantly low penalty pressure (dark markers), while additional instances extend into moderate coverage (≈ 0.22–0.32) with similarly high objective potential. A small set of outliers departs from this band, exhibiting much lower objective potential (down to near 0–20) at moderate-to-high coverage (≈ 0.20–0.45), representing more challenging regimes where limited high-value content coincides with stronger temporal restriction. Marker shading highlights that higher penalty pressure is mainly concentrated among a few low-coverage cases, whereas most instances remain in the low-penalty region.

### 4.2 Instance space visualization

To visualize global instance heterogeneity across the many structural and temporal dimensions captured in this study, we construct a low-dimensional instance space using Instance Space Analysis (ISA) [8]. Rather than comparing algorithm performance, which is the typical focus of ISA, we concentrate on visualizing the distribution and diversity of our dataset. Since we have defined this problem for the first time, no existing benchmark is available; our dataset represents all the instances.

To assess the distribution and diversity of the dataset, we follow a systematic approach. First, we select a set of relevant features that capture the characteristics of our instances. These features are then pre-processed to ensure consistency and suitability for analysis. Next, we apply dimensionality reduction techniques to condense the features into two dimensions, enabling visualization. Finally, we plot the resulting two-dimensional representation to reveal the distribution and diversity patterns within the dataset.

The selected features represent the structural and behavioural characteristics of the instances. To derive these features, we utilized the parameters from the original dataset [2] that were used during instance generation. Rather than relying solely on raw parameters, we computed derived features that capture the global characteristics of the instances, providing more meaningful insights into their behaviour. Initially, we selected a larger feature set, but through experimentation identified and removed highly correlated features, retaining (see Table 2) only those that exhibited strong diversity and information content. Since most features displayed skewed distributions, we applied quantile transformation to normalize their ranges. This transformation ensured that no single feature dominated the analysis with extreme values, allowing for a balanced representation of instance characteristics.

**Table 2:** Feature metrics used for instance space analysis.

Metric	Description
Duration Tightness	Measures how restrictive *min_duration* is relative to actual program lengths. Computed as the ratio of programs that fail the minimum duration threshold. High values mean many programs are too short to schedule, tightening the solution space.
Genre Diversity	Quantifies genre variety using Shannon entropy. Low diversity (e.g., most programs are one genre) combined with strict *max_consecutive_genre* can make scheduling difficult or infeasible. Captures content heterogeneity independent of constraint values.
Priority Block Friction	Percentage of otherwise-schedulable programs forbidden by *priority_block* hard constraints. Computed by identifying programs on non-allowed channels during each priority window. High friction indicates major search-space reduction in those intervals.
Average Program Score	Mean of all program score values in the instance. Higher averages indicate richer reward potential and more high-quality options; low averages imply a sparse reward landscape.
Bonus Potential	Total achievable bonus from *time_preferences*, computed via overlap between preferred time windows and programs matching *preferred_genre*. Interprets the maximum soft-constraint reward available.
Availability Density	Ratio of total program minutes to theoretical capacity:
Horizon Length	Total scheduling window in minutes: *closing_time* - *opening_time*. Longer horizons increase instance size, state space, and temporal dependency chains.
Avg Program Duration	Mean program duration (minutes). High averages mean fewer but longer scheduling blocks; low averages mean many short programs and more frequent decisions.
Std Program Duration	Standard deviation of program durations. High values indicate mixed short/long programs and uneven blocks; low values indicate uniform durations that simplify alignment.
Avg Concurrent Choices	Average number of overlapping programs available at any timestep across all channels (branching factor). High concurrency sharply increases combinatorial complexity; low concurrency indicates more constrained, easier instances.

We then embed these normalized feature vectors using three complementary dimensionality-reduction techniques: Principal Component Analysis (PCA) [9], t-Distributed Stochastic Neighbour Embedding (t-SNE) [10], and Uniform Manifold Approximation and Projection (UMAP) [11]. PCA provides a linear projection that highlights dominant variance structure, t-SNE emphasizes local neighbourhood relationships in a non-linear fashion, and UMAP preserves both local and global manifold structure in a non-linear low-dimensional layout.

For PCA, we retained the first two principal components, which together explained 76.8 % of the total variance, indicating that they capture the underlying structure of the data well. For t-SNE, we experimented with perplexity values ranging from 1 to 100 to identify the optimal configuration. By analysing the Kullback-Leibler (KL) [12] divergence and the resulting visualizations for each perplexity value, we determined that a perplexity of 96 yielded the lowest KL divergence. For UMAP, we systematically explored combinations of *n_neighbors* (5, 10, 15, 30, 50, 100) and *min_distance* (0.0, 0.1, 0.25, 0.5, 0.8, 1.0) parameters. Through evaluation of trustworthiness and silhouette scores, we found that *n_neighbors* = 10 and *min_distance* = 0.1 provided the best trade-off, achieving a trustworthiness score of 0.96 and a silhouette score of 0.16.

**Fig. 13:**
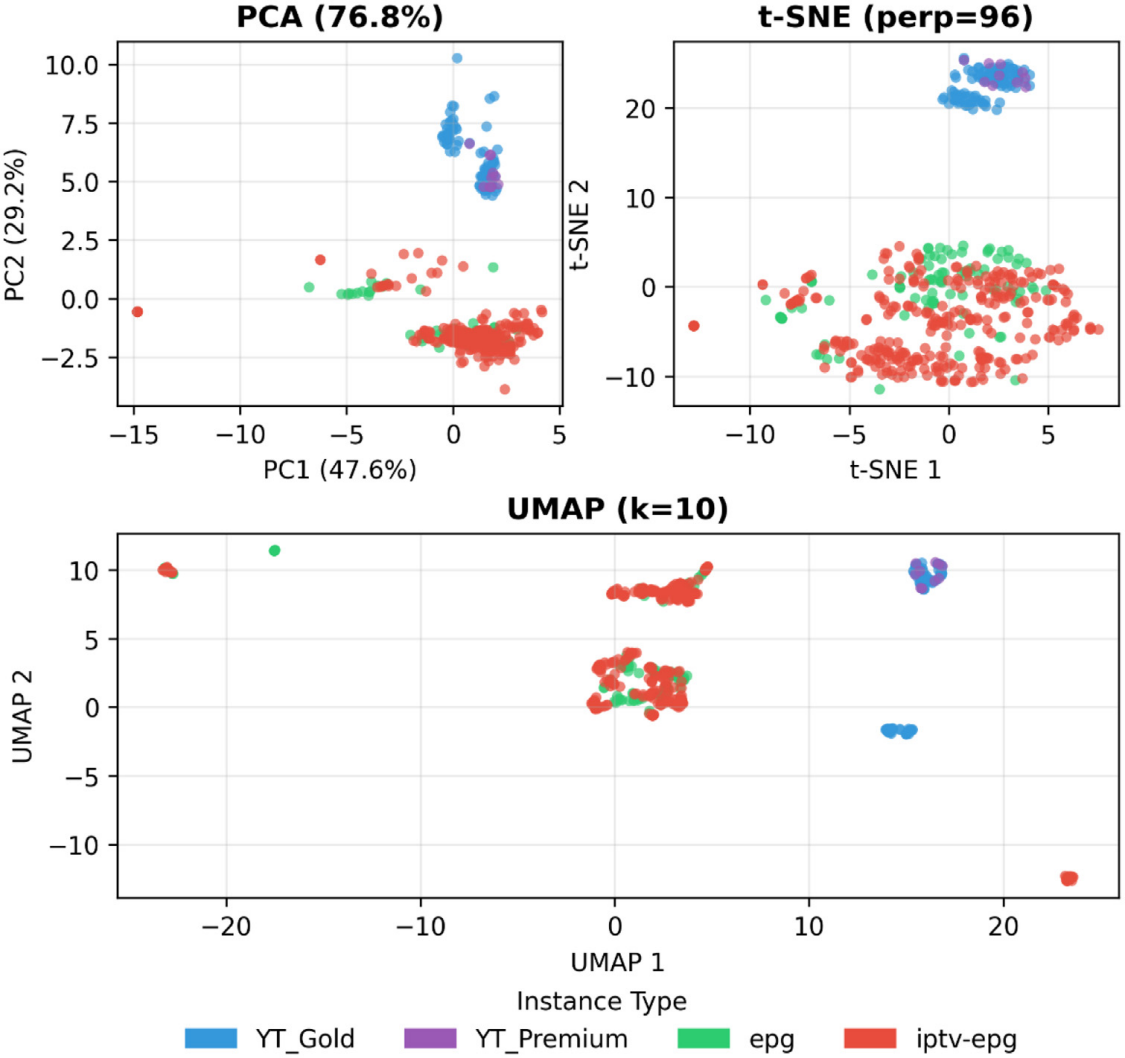
Instance space visualization via PCA, t-SNE, and UMAP, showing source-specific clustering.

Fig. 13 presents the resulting two-dimensional embeddings from all three techniques, revealing several consistent patterns across the instance space. Most notably, YouTube instances (YT Gold and YT Premium) occupy distinctly different regions compared to EPG-based instances (epg and iptv-epg), a separation that remains consistent across all three dimensionality reduction methods. Within the YouTube instances, YT Gold exhibits a bimodal distribution, spanning two distinct clusters, while YT Premium concentrates primarily in only one of these clusters - a pattern observable in all three visualizations.

The EPG-based instances display more overlap, with both epg and iptv-epg types occupying similar regions of the instance space across all three plots. However, closer examination reveals that the two major EPG clusters show differing composition: one cluster contains a higher concentration of epg instances, while the other is dominated by iptv-epg instances. A small number of EPG outliers appear consistently across the three visualizations, positioned in the peripheral regions of the instance space (lower-left in PCA and t-SNE, lower-right and upper-left in UMAP).

Notably, all three plots reveal sparsely populated or empty regions within the instance space, suggesting opportunities for future dataset enrichment through targeted synthetic instance generation in these underrepresented areas.

## 5 Experimental Design, Materials and Methods

### 5.1 Use of source data, permissions, and legal basis

The dataset was constructed from (i) publicly accessible Electronic Program Guide feeds provided via machine-readable XMLTV endpoints (iptv-epg.org and epg.pw) and (ii) publicly visible YouTube metadata retrieved exclusively through the official YouTube Data API v3.

The dataset contains only program-level scheduling metadata required for benchmark instance construction, including program identifiers, titles, channel identifiers, and scheduled start and end timestamps.

EPG data were accessed through publicly available XMLTV endpoints intended for program-guide consumption. The acquisition pipeline parses 〈channel〉 and 〈programme〉 elements and transforms the extracted metadata into a standardized JSON schema for research benchmarking purposes. The dataset does not redistribute the original XMLTV feeds, platform services, or media content. Instead, it contains transformed, date-filtered, and normalized schedule metadata enriched with derived attributes required for reproducible scheduling experiments.

The use of IPTV-EPG.org data is subject to the provider’s publicly available terms, notices, and usage conditions applicable at the time of access. The dataset does not reproduce the IPTV-EPG.org website, infrastructure, services, or complete feed archives. The redistributed material consists solely of structured, transformed schedule metadata necessary for research benchmarking.

The use of EPG.PW data is subject to the provider’s published Terms of Service [13]. The dataset does not reproduce the EPG.PW website, infrastructure, or services, and does not republish complete feed archives. The redistributed material consists solely of structured, transformed schedule metadata necessary for research benchmarking.

YouTube metadata were retrieved exclusively through the official YouTube Data API v3 in accordance with the YouTube API Services Terms of Service [14] and the YouTube API Services Developer Policies [15]. The dataset does not reproduce YouTube services, media content, platform functionality, or complete API responses. The redistributed material consists solely of structured, transformed scheduling metadata derived from publicly visible API fields necessary for reproducible scheduling experiments.

### 5.2 Data acquisition and preprocessing

**Fig. 14:**
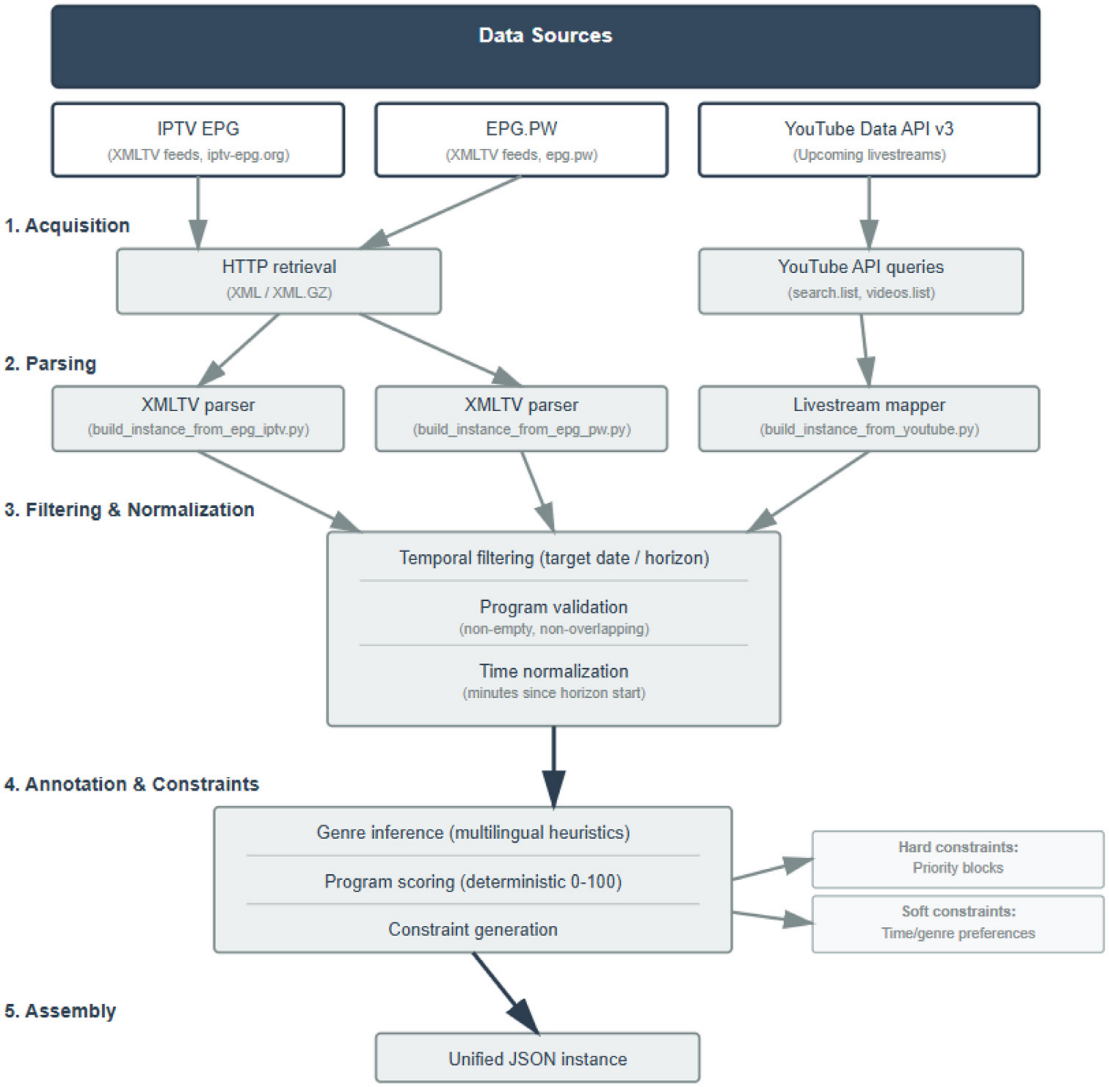
Data acquisition and instance construction workflow.

Scheduling instances were gathered from three publicly accessible sources: IPTV EPG XMLTV feeds obtained from iptv-epg.org, XMLTV feeds obtained from epg.pw, and upcoming YouTube livestream metadata collected through the YouTube Data API v3 (*search.list, videos.list*). The overall acquisition and construction workflow is summarized in Fig. 14. All processing was implemented in Python using deterministic scripts.

For the two broadcast-derived sources (IPTV EPG and EPG.PW), XMLTV files were retrieved over HTTP in XML or compressed XML.GZ format and parsed into program interval records. For YouTube, API queries were used to obtain scheduled livestream entries and their associated metadata (e.g., channel identifiers, titles, planned start times, and durations when available). Source-specific parsers were used to map raw records into a common internal representation (build_instance_from_epg_iptv.py, build_instance_from_epg_pw.py, and build_instance_from_youtube.py), extracting channel identifiers and program start–end intervals in a consistent format.

All instances then passed through the same filtering and normalization pipeline. Programs were first temporally filtered to the target date and a fixed scheduling horizon. Entries failing basic validity checks (e.g., missing timestamps, empty intervals, or inconsistent start/end times) were removed. Time values were then normalized by converting timestamps into integer minutes relative to the start of the horizon, enabling uniform processing across heterogeneous sources.

After normalization, instances were annotated and enriched with constraints. Program genres were inferred using multilingual heuristic rules [16] applied to available metadata. Program scores were computed deterministically on a 0–100 scale. Hard constraints were encoded as priority blocks, defined as time windows during which only specified subsets of channels are permitted. Soft constraints were modelled as time preferences represented by additive bonus intervals.

Finally, each instance was assembled into a unified JSON representation and validated against a formal schema (instance_schema.json). The dataset outputs are organized by source in separate directories (Instances_IPTV/, Instances_PW/, and Instances_YouTube/, including Gold/Premium subsets), with all generated files produced through deterministic Python pipelines.

## Limitations

The instances were compiled from multiple upstream schedule sources (IPTV EPG feeds, provider-based EPG feeds, and YouTube “upcoming” livestream metadata) and harmonized into a unified JSON representation. Because the EPG-based data were collected over a limited date range, the dataset represents a short-term snapshot and may not capture seasonal or long-term variation in programming patterns. As the dataset relies on public and aggregated feeds, metadata quality varies across sources and regions; program labels such as genre and category, along with other descriptive fields, may be missing, inconsistent, or differently defined by upstream providers. The YouTube subset is derived from “upcoming” livestream listings and mapped into television-like channels and programs; these events can be rescheduled, modified, or cancelled after collection, so the captured schedules reflect the state at the time of retrieval rather than finalized broadcast schedules. Automatic validation focuses on structural consistency (e.g., well-formed time windows and non-overlapping program intervals) and does not correct semantic inaccuracies inherited from upstream metadata.

## Ethics Statement

The authors confirm that this work does not involve human subjects or animal experiments. The dataset was compiled from publicly accessible electronic program guide (EPG) endpoints and publicly visible metadata for upcoming YouTube livestreams. Data collection and redistribution were conducted with attention to the applicable terms and policies of each upstream source; in particular, YouTube data were collected using permitted access methods (e.g., official APIs where required) and without scraping YouTube applications. The dataset contains program-level schedule metadata (e.g., titles, times, channels) and does not include personal or sensitive user data.

YouTube metadata were retrieved only via the official YouTube Data API v3 in accordance with the YouTube API Services Terms of Service and Developer Policies [15]. Storage and redistribution of API-derived metadata are intended to follow YouTube’s rules on caching/retention, including requirements to refresh or delete stored resource metadata within the time limits defined in the Developer Policies.

## CRediT Author Statement

**Kadri Sylejmani:** Conceptualization, Methodology, Validation, Formal analysis, Writing- Original draft preparation; **Shefket Bylygbashi:** Software, Data curation, Writing- Original draft preparation, Visualization; **Uran Lajçi:** Software, Visualization, Investigation, Writing- Original draft preparation; **Zenun Kastrati:** Writing- Reviewing and Editing, Validation.

## Data Availability

Zenodo - Open repository for EU-funded research outputs from Horizon Europe, Euratom, and earlier Framework Programmes.Smart TV Scheduling Dataset: Multi-Source Benchmark Instances for Constraint-Based Media Planning (Original data).
